# Dietary supplement use among health care professionals enrolled in an online curriculum on herbs and dietary supplements

**DOI:** 10.1186/1472-6882-6-21

**Published:** 2006-06-12

**Authors:** Paula Gardiner, Charles Woods, Kathi J Kemper

**Affiliations:** 1 Division for Research and Education in Complementary and Integrative Medical Therapies, Harvard Medical School, Boston, Massachusetts, USA; 2 Department of Pediatrics and Public Health Sciences, Wake Forest University School of Medicine, Winston-Salem, North Carolina, USA; 3 Department of Pediatrics, Public Health Sciences and Family and Community Medicine, Wake Forest University School of Medicine, Winston-Salem, North Carolina, USA; 4 Division for Research and Education in Complementary and Integrative Medical Therapies, Osher Institute, Harvard Medical School, 401 Park Drive Suite 22A-West, Boston, MA 02215

## Abstract

**Background:**

Although many health care professionals (HCPs) in the United States have been educated about and recommend dietary supplements, little is known about their personal use of dietary supplements and factors associated with their use.

**Methods:**

We surveyed HCPs at the point of their enrollment in an on-line course about dietary supplements between September, 2004 and May, 2005. We used multivariable logistic regression to analyze demographic and practice factors associated with use of dietary supplements.

**Results:**

Of the 1249 health care professionals surveyed, 81 % reported having used a vitamin, mineral, or other non-herbal dietary supplements in the last week. Use varied by profession with highest rates among nurses (88%), physician assistants or nurse practitioners (84 %) and the lowest rates among pharmacists (66%) and trainees (72%). The most frequently used supplements were multivitamins (60%), calcium (40%), vitamin B (31%), vitamin C (30%), and fish oil (24%). Factors associated with higher supplement use were older age, female, high knowledge of dietary supplements, and discussing dietary supplements with patients. In our adjusted model, nurses were more likely than other professionals to use a multivitamin and students were more likely to use calcium.

**Conclusion:**

Among HCPs enrolled in an on-line course about dietary supplements, women, older clinicians, those with higher knowledge and those who talk with patients about dietary supplements had higher use of dietary supplements. Additional research is necessary to understand the impact of professionals' personal use of dietary supplements on communication with patients about them.

## Background

Vitamins, minerals and dietary supplements are among the most commonly used complementary or alternative medical (CAM) therapies in the United States [1,2]. Health care professional's personal use of dietary supplements is of interest for several reasons. Patients ask health care professionals (HCP) to provide advice about vitamins, minerals, and other dietary supplements, and a health care professional's personal health habits may affect whether they will recommend a dietary supplement [3]. In health care professional training programs, many health care professionals learn about the role that nutrition and dietary supplements play in the prevention and treatment of chronic diseases such as osteoporosis, heart disease, cancer, and neural tube defects [4-7]. For example, federal and national guidelines have recommended that women take calcium and vitamin D for the prevention of osteoporosis; [8-12] that patients take fish oil for the prevention of heart disease and hypertension; [13-15] that women of child-bearing age take folate for the prevention of neutral tube defects in infants [16-18] and people over age 50 consume vitamin B12 [17].

Few studies have looked at the extent to which health care professionals (HCPs) follow these dietary supplement guidelines personally, and if their own dietary supplement use influences their willingness recommend and communicate with patients about dietary supplements.

Additionally, little is known about the relationship between HCP's use and demographic or professional characteristics such as age, gender, professional group, knowledge or confidence about recommending dietary supplements [19-23].

Using data from a cross sectional survey of health care professionals enrolled in an online curriculum about dietary supplements, we examined overall prevalence of dietary supplement use by profession; personal and professional factors associated with supplement use; and the relationship between specific supplements used and knowledge, confidence, and self reported communication with patients about dietary supplements.

We hypothesized that as it is in the general public, dietary supplement use would be more common among female health care professionals and middle-aged or older health care professionals. Further, we hypothesized that personal use of vitamins, minerals and dietary supplements would be significantly associated with greater knowledge about, greater confidence in talking about, and more frequent communication with patients about herbs and dietary supplements.

## Methods

This was a cross sectional survey of all participants prior to the start of a web based dietary supplements curriculum. Questions included participants' personal use of vitamins, minerals, and other dietary supplements excluding herbs, demographic and professional characteristics, and questions to assess knowledge about dietary supplements. We excluded herbs in this analysis, because herbs are not traditionally taught about in health care professional schools for treatment and prevention of medical conditions, thus we would not expect for health care practitioners to have professional knowledge of them. Herb use among these professionals has been described in a separate report [24].

### Recruitment

The recruitment protocol for our on-line dietary supplement education trial has been described previously [25]. Overall, from 2004 to 2005, we sent approximately 59,000 email announcements and 19,500 flyers to medical schools, health professional groups and continuing medical education programs nationwide [25]. Respondents were recruited to a webpage, and registration occurred on-line through the North Carolina Northwest Area Health Education Center (NW AHEC).

Following registration, participants completed the baseline questionnaire on-line. Participants were eligible for our analysis if they were in one of the following professional groups: physicians; clinical nurses; physician assistants or nurse practitioners (NP or PA); pharmacists; dietitians; or trainees in one of these health professions. This resulted in a total sample of 1249 eligible participants.

### Survey instrument

The survey instrument used in this project was based on a previous pilot study and has been described elsewhere [25]. In addition to demographic data, participants were asked about their profession and if they had seen any patients in the prior 30 days. Personal use of dietary supplements in the last week was evaluated with a list of approximately 59 herbs and 27 vitamins, minerals and other dietary supplements.

*Knowledge scores *were assessed with 28 true/false and multiple choice questions about commonly used dietary supplements. Scores could range from 0 to 100% correct. The knowledge questions included inquires on the use and safety of commonly used herbs and dietary supplements such as vitamins/minerals, folate, chromium, vitamin D; and other dietary supplements such as creatine, glucosamine, Coenzyme Q10, and fish oil. [see Additional file 1] Knowledge scores were divided into three categories with scores (percent of questions answered correctly), with scores ≤ 58% correct considered low (N = 364), 59–69% as medium (N = 483), and ≥70% as high (N = 402).

A *confidence scale *was designed to measure respondent's confidence in answering patient questions and making recommendations about dietary supplements to patients. The score was derived from responses to 19 Likert-type questions (strongly disagree, disagree, neutral/not sure, agree, strongly agree) such as "I feel confident responding to patients' questions about HDS."; "I can warn patients about side effects of commonly used herbs and dietary supplements." [see Additional file 1] Each item was scored 1 (strongly disagree) to 5 (strongly agree), with a minimum score of 19 and maximum of 95. The Cronbach alpha reliability statistic was 0.96 for the scale. The confidence score was divided into approximate quartiles: low (N = 387), medium (N = 289), high (N = 275), and very high (N = 297).

*Communication scale: *The subset of respondents (N = 1176) who reported having seen patients in the 30 days prior to the survey were asked about communication with patients about dietary supplement use. (e.g., "In the past 30 days, in what percentage of your clinical encounters have you discussed with a patient or family the use of herbs and dietary supplements?"). Respondents marked the percentage of times they discussed herbs and dietary supplements on a scale of 0 to 100%. Potential scores could range from 0 to 10. This response was dichotomized (yes or no) into a variable "discussed with patients".

### Statistical analysis

Descriptive statistics were used to examine the prevalence of dietary supplement use and the most common dietary supplements. Chi-square tests were used to compare proportions of characteristics in individual dietary supplement users versus non-users. Logistic regression analysis was used to determine demographic and clinical practice characteristics associated with use of vitamin, mineral and other dietary supplements reported by as used by ≥15 % of the respondents. Based on prior analyses and the bivariate results, eight variables were selected for multivariable analysis in single step modeling: age, gender, race/ethnicity, geographic residence (North Carolina versus elsewhere), profession, knowledge score, confidence score, and the "discussed with patients" variable [25]. Among the professions, physician assistants and nurse practitioners were grouped together due to their similar degree of advanced training and ability to prescribe medications. All analyses were performed using SAS-9 [26]. This study was approved as "exempt" as an educational research project by the Wake Forest University School of Medicine Institutional Review Board.

## Results

Of the 1249 health care professionals surveyed, 21% were physicians, 16% were clinical nurses, 6% were nurse practitioners or physician assistants, 11% were dieticians, 3% were pharmacists, 37% were students, and 7% were residents or fellows. The average age was 40.3 ± 12.9 years, 25% percent were men; 83% were Caucasian, 8% were Asian, 5 % were African American, and less than 1% were Native American/Alaskan Native. Forty seven percent of respondents lived outside North Carolina. Among health care professionals who had seen patients in the past month, 57% reported having discussed dietary supplements with patients at least once. The average knowledge score was 65.8 ± 10.7% correct.

Overall, 81 % reported having used a vitamin mineral or other dietary supplements, 79% reported using a vitamin or mineral, and 35% reported using another non-herb dietary supplement in the last week. The most commonly used were multivitamins (60%), calcium (40%), vitamin B (31%), vitamin C (30%), fish oil (24%), vitamin E (23%), vitamin D and folate (15% each) (Table 1).

**Table 1:** Frequency of vitamin, mineral and other dietary supplements

	N = 1249	
**Dietary supplement**	N	% of respondents using

**Any Vitamin**	942	75
Multivitamins	762	60
Vitamin B	382	31
C Vitamin	373	30
E Vitamin	286	23
Folate model	187	15.
D Vitamin	192	15
Niacin, B3	95	8
Vitamin K	74	6
Lutein	49	4
**Any Mineral**	567	45
Calcium	495	40
Magnesium	158	13
Iron	122	10
Chromium	64	5
**Other Dietary Supplement**	438	35
Fish oil	302	24
Glucosamine	152	12
Co Q 10	128	10
Alpha linoleic acid	75	6
MSM – in plants	61	5
DHEA	32	3
Melatonin	41	3
SAM-e	17	1
Creatine	8	.6

With regard to each individual profession, use was highest among nurses (88%), nurse practitioners and physician assistants s (84%), and lowest among pharmacists (66%) and trainees (72%) (P < .01). (Table 2)

**Table 2:** Respondents baseline characteristics for commonly used vitamins minerals and other dietary supplements *

**Characteristic**	**Total N (%)**	**Multi vitamin**	**Calcium**	**Vitamin B**	**Vitamin C**	**Fish oil**	**Vitamin E**	**Vitamin D**	**Folate**
N	1249	754	495	382	373	295	286	192	187
		60%	40%	31%	30%	24%	23%	15%	15%
**Age**									
≤30	406 (32.5)	33	31	22	28	12	15	11	10
31–40	202 (16.2)	16	38	23	26	24	14	12	12
41–50	328 (26.3)	25	43	32	27	27	27	15	15
>50	313 (25.1)	25	49	45	38	35	35	24	23
		P = .85	P < .001	P < .001	P < .001	P < .001	P < .001	P < .001	P < .001
**Gender**									
Male	316 (25)	22	19	28	31	25	23	10	11
Female	933 (75)	78	47	31	30	23	23	17	16
		P < .001	P < .001	P = .17	P = .70	p = .50	P = .96	P = .01	P = .04
**Race/Ethnic**									
African American	57 (4.6)	4	16	32	26	18	26	11	11
Asian/P.I.	95 (7.6)	7	32	16	19	16	9	12	8
Caucasian	1035 (82.9)	84	42	32	31	24	24	16	16
Native American	3 (0.2)	4	67	33	33	0	33	33	33
Declined	59 (4.7)	.3	27	34	36	29	25	20	12
		P = .711	P < .001	P = .03	P = .13	P = .15	P = .03	P = .41	P = .19
**Professional Group**									
Physician	256 (20.5)	18	34	33	29	32	26	18	19
Dietician	137 (10.97)	11	54	6	17	24	16	12	12
Nurse	203 (16.3)	19	48	45	34	28	36	19	19
PA & NP	73 (5.8)	6	51	45	38	37	26	22	19
Pharmacist	41 (3.3)	3	46	20	24	32	20	15	12
Student	458 (36.7)	37	35	28	32	14	17	13	12
Residents & fellows	81 (6.5)	6	27	19	23	20	14	9	14
		P = .02	P < .001	P < .001	P < .001	P < .001	P < .001	P = .08	P = .09
**NC resident**									
Yes	587 (47)	45	43	30	28	23	24	16	14
No	662 (53)	55	36	31	32	24	22	15	16
		P = .15	P = .01	P = .85	P = .13	P = .54	P = .37	P = .78	p = .35
**Knowledge Scores**	Average 65.8 ± 10.7								
High	402 (32)	35	52	38	32	36	26	19	22
Medium	483 (39)	39	37	31	30	23	23	14	14
Low	364 (29)	26	30	21	28	11	19	13	8
		P = .007	P < .001	P < .001	P = .47	P < .001	P = .07	P = .07	P < .001
**Confidence Score**									
Low	387 (31)	30	27	24	29	22	26	27	26
Medium	289 (23)	25	23	25	26	23	26	24	23
High	275 (22)	22	23	25	23	24	21	20	24
Extremely high	297 (24)	23	27	26	22	31	26	29	28
		P = .12	P = .08	P = .01	P = .30	P < .001	P = .09	P = .21	P = .29
Discuss HDS with Patients*									
Yes	667 (57)	56	43	34	30	30	25	18	19
No	510 (43)	44	35	25	29	16	20	11	11
		P = .55	P = .002	P = .001	P = .70	P < .001	P = .04	P < .001	P < .001

*(15 % or > of the sample size).

Eight variables were selected for multivariable logistic regression analysis in single step modeling for adjusted association with uses of multivitamins, vitamins D, C, E, B, calcium, folate, and fish oil: age, gender, race/ethnicity, geographic residence (North Carolina versus elsewhere), profession, knowledge score, confidence score, and the "discussed with patients" variable. These results are provided in Table 3. Modeling was performed with (N = 1176) and without (N = 1249) the "discuss with patients" variable. Since results were highly similar with both, the results presented include this variable.

**Table 3:** Amongst health care professionals, factors associated with individual use of vitamins and minerals; adjusted multivariate logistic regression***

**Characteristic**	**Multivit**	**Calcium**	**Vit B**	**Vit C**	**Vit E**	**Vit D**	**Folate**	**Fish oil**
**Gender**								
Male *	1.0	1.0	1.0	1.0	1.0	1.0	1.0	1.0
Females	1.3 (.93–1.7)	3.4 (2.4–4.8)	2.3 (1.4–3.8)	.99 (.74–1.4)	.88 (.60–1.3)	2.3 (1.4–.3.8)	1.8 (1.2–2.9)	.88 (.60–1.3)
**Professional Group**								
Physician *	1.0	1.0	1.0	1.0	1.0	1.0	1.0	1.0
Nutritionist	1.3 (.80–2.1)	1.6 (.99–2.6)	.47 (.24 .92)	.56 (.32.99)	.67 (.37–1.2)	.47 (.24–.92)	.56 (.29–1.1)	.94 (.55–2.2)
Nurse	2.3 (1.4–3.6)	1.5 (.96–2.5)	.73 (.41–1.3)	.1.2 (.75–2.0)	1.6 (1.0–2.7)	.73 (.41–1.3)	1.1 (.61–1.9)	1.3 (.81–2.2)
PA and NP	1.1 (.64–2.0)	1.1 (.63–2.0)	.78 (.39–1.6)	1.4 (.79–2.5)	1.6 (.87–2.9)	.78 (.39–1.6)	.73 (.35–1.5)	1.3 (.73 –2.4)
Pharmacist	.88 (.43–1.8)	1.6 (0.78–3.5)	.77 (.29–2.1)	.91 (.40 2.1)	.81 (.33 2.0)	.77 (.29–2.1)	.67 (.24–1.9)	.99 (.43–2.2)
Student	1.4 (.87–2.2)	1.7 (1.05–2.7)	.96 (.52–1.8)	1.9 (1.2–3.1)	1.1 (.66–2.0)	.96 (.52–1.8)	1.2 (.64–2.2)	1.0 (.60–1.7)
Resident	.83 (.47–1.4)	.79 (0.42–1.5)	.35 (.12–.95)	.89 (.47–1.7)	.57 (.47–1.7)	.35 (.12–.96)	.82 (.36–1.8)	.74 (.37–1.5)
**Age (years)**								
≤30*	1	1.0	1	1	1	1		1
31–40	.88 (.58–1.3)	1.1 (0.72–1.7)	.75 (.40–1.4)	1.1 (.70–1.7)	.93 (.53–1.6)	.75 (.40–1.4)	.93 (.50–1.7)	1.5 (.89–2.5)
41–50	.78 (.50–1.1)	1.4 (0.9– 2.1)	1.1 (.58–1.9)	1.5 (.93–2.3)	2.0 (1.2–3.3)	1.1 (.58–1.9)	1.1 (.59–2.0)	1.6 (.95–2.7)
>50	.82 (.53–1.3)	2.0 (1.3–3.1)	2.1 (1.1–3.8)	2.2 (1.4–3.5)	2.4 (1.4–4.1)	2.1 (1.1–3.8)	2.3 (1.3–4.1)	2.5 (1.5–4.2)
**Knowledge Scores**								
Low*	1.0	1.0	1.0	1.0	1.0	1.0	1.0	1.0
Medium	1.4 (1.04 – 1.9)	1.3 (.97–1.9)	1.1 (.68 1.7)	1.7 (.84 1.6)	1.2 (.83 1.8)	1.1 (.68 1.7)	1.7 (1.05–2.7)	1.9 (1.3–3.9)
High	2.0 (1.4–2.8)	2.7 (1.9–3.9)	1.4 (.87–2.2)	1.4 (.97–2.0)	1.4 (.93–2.1)	1.4 (.87–2.2)	2.7 (1.6–4.4)	3.4 (2.2 –5.3)
**Clinical Confidence**								
Low	1.0	1.0	1.0	1.0	1.0	1.0	1.0	1.0
Medium	1.4 (1.0– 1.9)	1.1 (.76–1.5)	1.2 (.77–1.9)	1.3 (.90–1.8)	1.4 (.97–2.1)	1.2 (.77–1.9)	1.02 (.64–1.6)	1.3 (.88–2.0)
High	1.1 (.76–1.5)	.98 (.69–1.4)	.95 (.59–1.5)	1.2 (.82–1.7)	1.1 (.72–1.6)	.96 (.60–1.6)	1.05 (.66–1.7)	1.4 (.90–2.0)
Extremely high	.96 (.68–1.4)	1.1 (.75–1.6)	1.3 (.80–2.1)	1.0 (.68–1.5)	1.3 (.84–1.9)	1.3 (.80–2.1)	1.2 (.74–1.9)	1.5 (1.0–2.3)
**Discuss with patients HDS**								
No *	1.0	1.0	1.0	1.0	1.0	1.0	1.0	1.0
Yes	.97 (.73–1.3)	1.34 (1.0–1.8)	1.7 (1.2–2.6)	1.1 (.81–1.5)	1.1 (.78–1.5)	1.7 (1.2–2.6)	1.7 (1.1–2.5)	1.4 (.97–1.9)

*The race/ethnicity variable did not show any positive association for other dietary supplement use in the multivariable analysis.

**The geographic variable showed a positive association (1.6 [1.2–2.2]) for fish oil and 1.3 [1.02 1.7] for multivitamin use in the multivariable analysis.

***Adjusted odds ratio with 95% confidence interval (N = 1176)

In adjusted models, the demographic factors most consistently associated with vitamin, mineral and other dietary supplement use were older age and being female. Nurses were more likely to use a multivitamin and students were more likely to use calcium.

In the adjusted model, there was a significant relationship between personal use of supplements (multivitamin, calcium, folate, and fish oil) and knowledge scores. There was a significant relationship between personal use of supplements (vitamin B and D, folate) and discussion with patients about dietary supplements. However, higher use of supplements was not associated with increased levels of confidence in talking with patients about dietary supplements.

## Discussion

In our study, supplement use varied by profession with highest rates among nurses, physician assistants and nurse practitioners. Dietary supplement use by the health care professionals in this study is similar to earlier studies of supplement use among dietitians and trainees [27-29], but higher than previous reports for pharmacists, physicians, and nurses [3,20,30-32]. Personal usage by the health care professionals in this study exceeds that of the general public in recent U. S. surveys. Our sample may have included highly motivated HCPs who use more HDS in general and may not be generalizable to the health care community at large[2,33,34]. The use of dietary supplements also may have increased in the intervals between these studies and ours. The types of dietary supplements reported as used by these health care professionals are similar to those used by the general public (e.g., multivitamins, calcium, and vitamin E) [2,35].

As in other studies, our multivariate regression analysis, showed that older age was positively associated with use of calcium, folate, vitamin B, vitamin D, fish oil, and female gender was positively associated with use of calcium, folate, vitamin D, and vitamin B [2,3]. Unlike earlier studies of the general public, our sample also reported high rates of using fish oil.

Our results are consistent with previous surveys of health care professionals showing a positive association between higher use, greater knowledge and increased communications with patients about dietary supplements [20,30,31,36,37]. Additional studies are needed to confirm or refute the hypothesis that clinicians with higher knowledge scores who use supplements themselves provide more knowledgeable guidance to patients about them, and to examine the impact of personal health behaviors on the quality of professional care.

After controlling for age and gender, nurses were more likely to use multivitamins and students were more likely to use calcium. More research is necessary to understand the impact of ones professional knowledge, and identity as a health care provider and how it influences health prevention behavior for oneself and one's patients.

Our respondents were self-selected because of their interest about vitamins, minerals and other dietary supplements; and they were also given an extensive list of commonly used supplements that might have enhanced recall and reporting. These two factors may have contributed to the somewhat surprisingly high rates of supplement use reported in this sample compared to earlier studies. Additionally, these results are based on self-report data rather than direct observation or interview which may over or under estimate true use. Data on professional recommendations, prescribing practices, personal use of prescribed and non-prescribed medications, daily doses of dietary supplements, and use of combination products were not collected as part of this study.

## Conclusion

In conclusion, this large study of diverse health professionals enrolled in an online dietary supplement curriculum confirms earlier studies showing higher use in women and older professionals. However, the results of this study are not generalizable to all health care professionals. Our analysis extends previous research by including a diverse group of health care professionals and by testing the association between personal use of supplements and professionals' knowledge, confidence and self-reported communication practices. It provides a strong basis for future studies to better understand the relationship between professional's use of dietary supplements and their quality of clinical care.

## Abbreviations

(HCP) health care professionals

## Competing interests

The author(s) declare that they have no competing interests.

## Authors' contributions

PG, KK, CW participated in the design of the study and PG, CW performed the statistical analysis. All authors read and approved the final manuscript.

## Pre-publication history

The pre-publication history for this paper can be accessed here:



## Supplementary Material

Additional File 1 Knowledge, attitude, and beliefs about herbs and dietary supplements questions. This document contains examples of original survey questions on knowledge, attitude, and beliefs about herbs and dietary supplements. We included thirteen true and false knowledge questions and nineteen questions measuring participants' overall confidence in dealing with herbs and supplements. Click here for file
