# Supplementary material for: Dietary supplement use among health care professionals enrolled in an online curriculum on herbs and dietary supplements
Source: BMC Complement Altern Med. 2006 Jun 12;6:21. doi: 10.1186/1472-6882-6-21 (PMC1526756; doi:10.1186/1472-6882-6-21)
Supplement: Additional File 1 — Knowledge, attitude, and beliefs about herbs and dietary supplements questions. This document contains examples of original survey questions on knowledge, attitude, and beliefs about herbs and dietary supplements. We included thirteen true and false knowledge questions and nineteen questions measuring participants' overall confidence in dealing with herbs and supplements. [file 1472-6882-6-21-S1.doc]

**Additional file 1**

**Knowledge, attitude and beliefs about herbs and dietary supplements questions**

|  | *Statement* | *True* | *False* |
| --- | --- | --- | --- |
| O_TFK1 | Saint Johns wort is typically used to treat depression. | X |  |
| O_TFK2 | Ephedra typically causes bradycardia (slow heart rate). |  | X |
| O_TFK3 | Saw palmetto is mainly used to treat prostate problems. | X |  |
| O_TFK4 | St. Johns wort can change serum concentrations of digoxin. | X |  |
| O_TFK5 | Many imported Chinese patent medicines are contaminated by pharmaceuticals, heavy metals, pesticides or herbicides. | X |  |
| O_TFK6 | Randomized controlled clinical trials have demonstrated that ginger is an effective remedy for nausea. | X |  |
| O_TFK7 | Three randomized controlled clinical trials have demonstrated that pycnogenol (pine bark extract) may be helpful in treating ADHD (attention deficit hyperactivity disorder). |  | X |
| O_TFK8 | More than 15% of adolescent boys undergoing sports physicals have reported using dietary supplements such as protein powders, amino acids, creatine or hormones. | X |  |
| O_TFK9 | Ginkgo can improve symptoms of dementia and cerebral insufficiency, but increase the risk of hemorrhage. | X |  |
| O_TFK10 | Folate can increase plasma homocysteine levels and increase the risk of heart attack. |  | X |
| O_TFK11 | Black cohosh has helped women with hot flashes, and is approved by the German Commission E to treat menopausal symptoms. | X |  |
| O_TFK12 | Licorice promotes cortisol breakdown and exacerbates inflammation. |  | X |
| O_TFK13 | Chromium has proven effective in normalizing serum glucose and HgbA1C levels in patients with Diabetes Mellitus. |  | X |

Check the ONE BEST answer that describes how you feel about each statement. There are no right or wrong answers in this section. These questions reflect participants’ overall confidence in dealing with herbs and supplements before taking the course. Your answers do not affect your course credit.

|  | *Statement* | *Strongly Agree* | *Agree* | *Neutral/ Not Sure* | *Disagree* | *Strongly Disagree* |
| --- | --- | --- | --- | --- | --- | --- |
| O_C1 | I feel confident responding to *patients’ questions* about H/DS. |  |  |  |  |  |
| O_C2 | I feel confident *initiating discussions* with patients about H/DS. |  |  |  |  |  |
| O_C3 | I know how to ask about which *brands and doses* patients are using of H/DS. |  |  |  |  |  |
| O_C4 | I can *warn patients* about *side effects* of commonly used H/DS. |  |  |  |  |  |
| O_C5 | I can *warn* patients about *interactions* between commonly used H/DS and medications |  |  |  |  |  |
| O_C6 | I can *provide* evidence-based information about H/DS to patients. |  |  |  |  |  |
| O_C7. | I can refer patients where to find information about the *quality* of different brands of H/DS. |  |  |  |  |  |
| O_C8 | I can tell my patients about the appropriate dose and duration to use H/DS. |  |  |  |  |  |
| O_C9 | I know where to *refer* patients for more information about H/DS |  |  |  |  |  |
| O_C10 | I know where *I* can turn for reliable information about H/DS. |  |  |  |  |  |
| O_C11 | I can readily *record* information about patients’ use of H’DS in the patient record. |  |  |  |  |  |
| O_C12 | I feel confident talking with *colleagues* about H/DS. |  |  |  |  |  |
| O_C13 | I know more about H/DS than many health care providers. |  |  |  |  |  |
| O_C14 | I know where and how to *report adverse effects* related to H/DS. |  |  |  |  |  |
| O_C15 | I could give a short lecture or demonstration to my colleagues about H/DS. |  |  |  |  |  |
| O_C16 | If a reporter or magazine writer called, I could answer questions about H/DS. |  |  |  |  |  |
| O_C17 | I can write a letter to the editor or a short review article about H/DS. |  |  |  |  |  |
| O_C18 | I can teach a high school science class about H/DS. |  |  |  |  |  |
| O_C19 | I can give a lecture about H/DS for students in my profession. |  |  |  |  |  |
